# Case Report: Successful Therapy of Spontaneously Occurring Canine Degenerative Lumbosacral Stenosis Using Autologous Adipose Tissue-Derived Mesenchymal Stem Cells

**DOI:** 10.3389/fvets.2021.732073

**Published:** 2021-09-23

**Authors:** Janko Mrkovački, Sanja Srzentić Dražilov, Vesna Spasovski, Amira Fazlagić, Sonja Pavlović, Gordana Nikčević

**Affiliations:** ^1^Stem Art Ltd., Belgrade, Serbia; ^2^Laboratory for Molecular Biomedicine, Institute of Molecular Genetics and Genetic Engineering, University of Belgrade, Belgrade, Serbia; ^3^National Association for the Improvement and Development of Regenerative Medicine, Belgrade, Serbia

**Keywords:** canine (dog), degenerative lumbosacral stenosis, autologous AT-MSCs, minimally invasive treatment, regenerative medicine, case report

## Abstract

The management of degenerative lumbosacral stenosis (DLSS) in dogs usually requires aggressive, costly surgical treatments that may themselves present complications, while do not fully resolve the symptoms of the disease. In this study, the dog diagnosed with severe DLSS, with hind limb paresis, was treated using a new and least invasive treatment. Cultured autologous adipose tissue-derived mesenchymal stem cells (AT-MSCs) were injected bilaterally at the level of L7-S1, in the vicinity of the external aperture of the intervertebral foramen of DLSS patient. In the previously described treatments of spontaneous intervertebral disc degeneration in dogs, intradiscal injections of MSCs did not lead to positive effects. Here, we report a marked improvement in clinical outcome measures related to the ability of a dog to walk and trot, which were expressed by a numeric rating scale based on a veterinary assessment questionnaire. The improved status persisted throughout the observed time course of 4.5 years after the AT-MSC transplantation. To the best of our knowledge, this is the first case of successful therapy, with long-term positive effect, of spontaneously occurring canine DLSS using presented treatment that, we believe, represents a contribution to current knowledge in this field and may shape both animal and human DLSS treatment options.

## Introduction

Canine degenerative lumbosacral stenosis (DLSS) is a syndrome of low back pain that includes various levels of neurologic dysfunction, and it has been defined as an acquired narrowing of the vertebral duct, the vertebral aperture, or both, resulting in compressive radiculopathy of cauda equina (1, 2). The degeneration of the intervertebral disc (IVD) L7-S1 is thought to be the cause of DLSS that occurs as a result of prolonged stress, along with the activity and age of the animal. Namely, when the elastic and fibrous annulus fibrosus weakens, and the central, gelatinous nucleus pulposus loses its hydration, the Hansen type II protrusion of the disc occurs that leads to the loss of the intervertebral spacing (3). The loss of normal biomechanical properties of the disc and the resulting degenerative changes in the supporting soft tissue and bony structures at the lumbosacral junction are the main contributors to the compression of sacral and caudal nerve roots (3). It is important to note that dogs with a transitional vertebra have an increased risk of DLSS due to increased rotational force caused by a poor position and poor articulation of the asymmetric lumbosacral junction (3).

Current management of DLSS in dogs comprise either conservative pharmacological treatments that frequently lead to a poor response, or aggressive and expensive surgical strategies that may themselves present complications, while do not fully resolve the symptoms (3). Recently, intradiscal injections of mesenchymal stem cells (MSCs) have been investigated in naturally developed DLSS in dogs, but this therapeutic approach did not lead to improvement of the disease (4, 5). This report proposes a novel and least invasive strategy, the transplantation of autologous adipose tissue-derived MSCs (AT-MSCs) in the vicinity of the external aperture of the intervertebral foramen of DLSS patient instead of intradiscally. Our aim was to avoid the harsh microenvironment within the IVD, assuming that the proposed route of application would allow AT-MSCs to exhibit their most important characteristics—trophic and homing effects at their full potential.

## Case Presentation

A client-owned 12-year-old female blend dog was presented with a several-month history of walk with flexed knees and hips, reduced activity with pronounced abstaining from climbing and descending the stairs, and with occasional urine incontinence. The dog weighed 19 kg (4–5 kg more than average) and had not received any medication beforehand.

During general examination, the dog was found to be conscious, oriented in space, with a normal posture of the head. The dog repeatedly supported body with forelegs, while hind legs were kept down, on the side. The perineal region was wet, most likely from urine.

On orthopedic and neurological examinations (Table 1), hind limb paresis with a mild drift of lumbosacral part, which was in a slight kyphosis, was noted. When the animal was placed to stand up on the hind legs, disturbed proprioception was observed, and quickly the dog rested again. Deep and superficial sensibility, retrieval reflex, and patellar reflex were preserved, while the crossed extensor reflex was slightly reduced. The stiffness was detected during each knee flexion with audible crepitation, which indicated osteoarthritis (OA) of both knees. Regarding hip flexion and extension, nothing abnormal was detected. During palpation of the lumbosacral region, the dog expressed discomfort and pain. The lordosis test (performed in a lying position) was positive; the animal felt pain. The enlarged urinary bladder was detected by palpation, which was released by pressure. Perineal reflex was preserved. The tone of the tail was decreased. Since the dog had no pain except the one that was elicited during physical examination (PE), and since she had had several episodes of gastritis in the past, no analgesic therapy was prescribed. A short video from that period was provided by the owner (Supplementary Video S1).

**Table 1:** Comprehensive information on the results of physical examination at each indicated patient's visit.

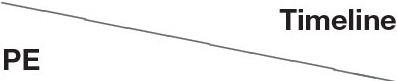		**Baseline**	**30 days**	**60 days**	**90 days**	**3 years**	**4.5 years**
**Orthopedic**							
L7-S1 region palpation		Expressed discomfort and pain	Less painful	No pain	UC	UC	UC
Lordosis test: lying position (LP) and standing position (SP)		Positive, animal felt pain (LP)	Positive but less than before the treatment (SP)	Negative (SP)	UC (SP)	UC (SP)	UC (SP)
Flexion, extension, adduction, abduction,	Knee	Stiffness and audible crepitation	UC	Less stiffness, audible crepitation	Mild stiffness at full flexion, with crepitation	UC	UC
circumduction	Hip	Normal	UC	UC	UC	UC	UC
**Neurologic**							
Proprioception		Disturbed	Less disturbed	Normal	UC	UC	UC
Deep and superficial sensibility		Preserved	UC	UC	UC	UC	UC
Retrieval reflex		Preserved	UC	UC	UC	UC	UC
Patellar reflex		Preserved	UC	UC	UC	UC	UC
Crossed extensor reflex		Slightly reduced	Normal	UC	UC	UC	UC
Perineal reflex		Preserved	UC	UC	UC	UC	UC
Tone of the tail		Decreased	Normal	UC	UC	UC	UC
Urinary bladder—palpation/pressure		Enlarged, released by pressure	Normal size, with sphincter tone	UC	UC	UC	UC
**General observations**		Hind limb paresis with a mild drift of lumbosacral part, which was in a slight kyphosis	Low hind limb paresis, other UC	Without paresis with slight drift of lumbosacral part	UC	Slight restraint during running	UC

*PE, physical examination; UC, unchanged*.

**Figure 1:**
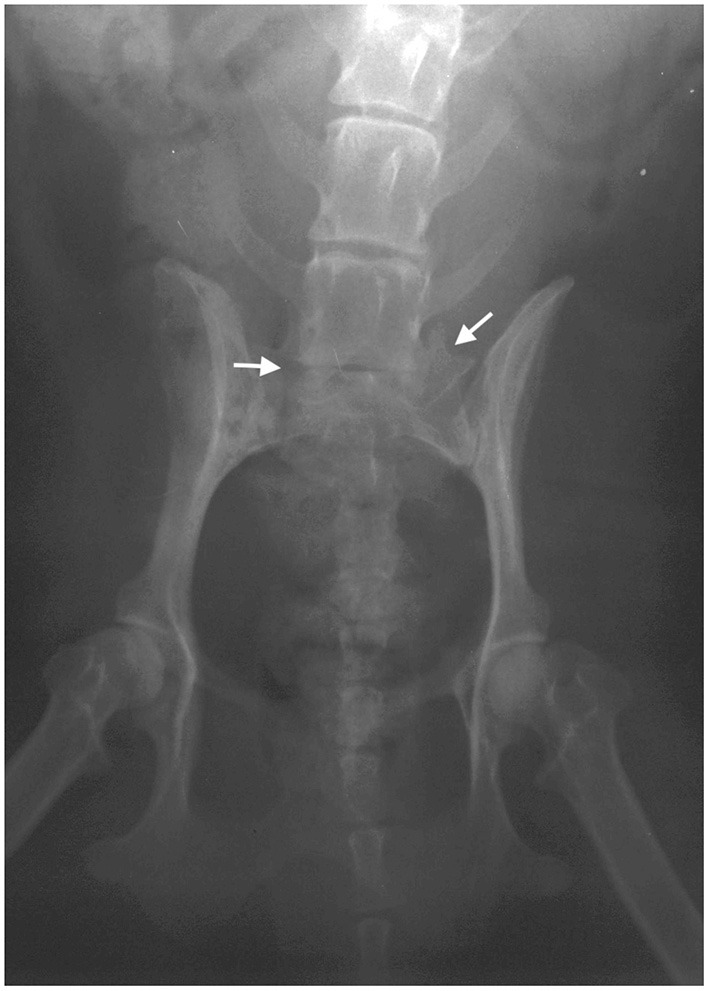
Radiograph image (X-ray) of the lumbosacral region. The image was taken in the ventrodorsal projection using the Atomscope radiograph machine (model 100pr type B, Auckland, New Zealand), with a collimator-to-film distance of 70 cm, exposure of 80 ms, and penetration power of 80 kV.

**Figure 2:**
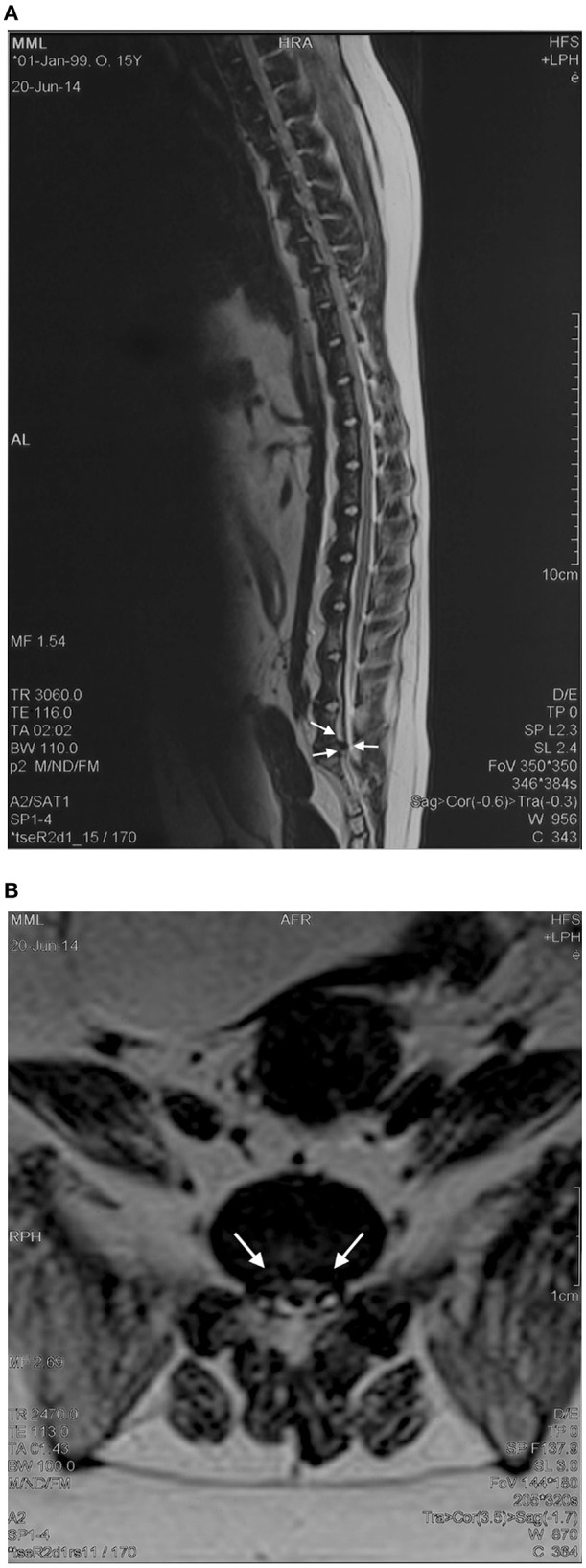
Representative magnetic resonance imaging (MRI) scans of the lumbosacral region. The T2-weighted sagittal **(A)** and transversal **(B)** images of the lumbosacral region are shown, and the intervertebral disc L7-S1 herniation is indicated by arrows.

Following described assessments, an X-ray and magnetic resonance imaging (MRI) were performed. The animal was anesthetized only during MRI due to her age and our technical limitation to complete these examinations consecutively. That is why the animal was poorly positioned on the radiograph image (Figure 1), but nevertheless, an asymmetric lumbosacral transitional vertebra with ossification on the left side was clearly seen (indicated by an oblique arrow), as well as a reduced intervertebral space L7-S1 (indicated by a horizontal arrow). On the sagittal MRI of the lumbosacral region (Figure 2A), the IVD L7-S1 was seen as protruded and hypointense compared with the bright signal from normally hydrated discs. The disc protrusion was also seen on the transversal MRI section (Figure 2B). Based on these results, as well as on orthopedic and neurological findings, this case was diagnosed as DLSS, accompanied with OA of both knees.

There is currently no consensus on treatment selection for dogs with DLSS, and available options most often involve aggressive surgical techniques (3). We decided to try a new and least invasive procedure, using cultured autologous adipose tissue-derived mesenchymal stem cells (AT-MSCs). These cells hold enormous potential as therapeutic agents *in vivo*, especially for regenerating damaged tissues in diseases of the locomotor system (6, 7).

The adipose tissue collection and AT-MSC therapy were approved and certified by the dog owner with signed informed consent. The AT collection was performed using anesthetics, and AT-MSC therapy was performed using sedatives, which excluded the pain, suffering, fear, and stress of the animal, following the standards of good veterinary practice, the guidelines of good laboratory practice, the European Parliament Directive, the Council of 22 September 2009 (2010/63/EU), and the European Convention for the protection of vertebrates intended for experimental and other scientific purposes (ETS 170).

Around 10 g of subcutaneous adipose tissue from the paralumbar region (on the borderline of the middle and caudal lateral abdominal region) was collected through the 5-cm-long incision while patient was under general anesthesia using medetomidine hydrochloride (Domitor, Orion Pharma, Espoo, Finland; 10 μg/kg IM) and Propofol (Diprivan, Astra Zeneca, Macclesfield, UK; 1.5 mg/kg IV).

The isolation, culture of AT-MSCs for the treatment, and differentiation of cells for control of the stemness were performed as previously described (8). A total of 91.8 × 10^6^ cells resuspended in 3 ml of PBS were transplanted as follows: paravertebrally at the level L7-S1, a 30.6 × 10^6^ cells were injected into each, left and right side with a 0.8 × 40-mm needle; while intraarticularly into each knee, a 15.3 × 10^6^ cells were injected with a 0.6 × 30-mm needle. During this procedure, the animal was sedated with medetomidine hydrochloride (Domitor, Orion Pharma, Espoo, Finland; 20 μg/kg IM). Following the application of AT-MSCs, the dog was not subjected to any physiotherapy treatment. Until the next PE, rest and restraint from physical activity under the owner's supervision were the only recommendations.

**Figure 3:**
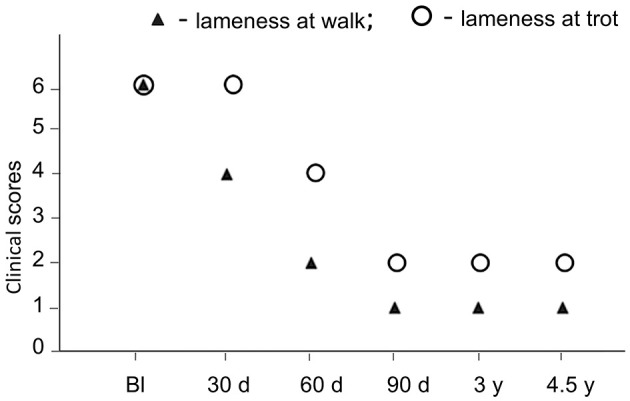
Timeline of the clinical course and effects of AT-MSC treatment on lameness of a dog at walk and trot during long-term follow-up. Bl, baseline; d, days; y, years. The numeric rating scale applied to evaluate clinical outcome in lameness at walk was as follows: 1, normal; 2, mild, sporadic; 3, persistent, weight bearing; 4, severe, occasionally weight bearing; 5, severe, non-weight bearing; 6, cannot walk without assistance; while for the lameness at trot was as follows: 1, normal; 2, mild, sporadic; 3, persistent, weight bearing; 4, severe, non-weight bearing; 5, cannot trot without stimulation; 6, cannot trot.

Clinical evaluation consisted of PE of the patient (Table 1). Also, the assessment for lameness at walk and trot, using a numeric rating scale, has been performed as previously described (8). Briefly, the clinical outcome measures were expressed by a numeric rating scale based on a veterinary assessment questionnaire. The scale ranged from 1 to 6, where 1 was marked as the best, normal condition (without the lameness) and 6 as the most severe (the animal cannot walk or trot). The evaluation was performed initially, upon admission of the patient (baseline) and at specified intervals after the treatment with AT-MSCs, supplemented with assessments of the owner (Figure 3). There were no detected adverse reactions to the described treatment.

As it is presented in Table 1, at first check-up performed 30 days after the treatment, the dog walked with stiff hind legs, occasionally weight bearing. Palpation of the lumbosacral region was less painful. The lordosis test (performed in a standing position) was positive but less than before the treatment. Stiffness during the flexion of both knees still existed, with audible crackling. The urinary bladder was of normal size and did not leak under the pressure. The tail had a normal tone. According to the owner, the dog was walking but with difficulties, could not run, turning was performed with an effort to maintain the balance of the backside, had problems when trying to get up and lie down, and it was difficult for the animal to take a position to urinate.

After 60 days of AT-MSC injection, the animal moved normally without the stiffness of the hind limbs. The knee stiffness was lesser than during previous examination. According to the owner, getting up and lying down was easier for the dog, and the position to urinate was almost normal.

Ninety days post-AT-MSC treatment, the animal functioned normally, with a desire to run and play, albeit cautiously. The mild stiffness of both knees remained at full flexion, with crepitation.

Once a year, the owner informed the veterinarian about the condition of the dog, which was unchanged compared with the examination that was performed on the 90th day after the AT-MSC treatment.

After 3 years posttreatment, the animal was re-examined. It was found that the dog still felt well, without any problems during the movement or with the balance, and without pain throughout the lordosis test. Slight restraint was observed during running. At full flexion, the mild stiffness of both knees was still present but painless. Videos from that period were provided by the veterinarian (Supplementary Videos S2, S3).

The final follow-up was performed 4.5 years after the treatment. The dog was still feeling well, with signs of slightly slower walking and turning and a bit less willingness to climb the stairs. Video from that period was provided by the veterinarian (Supplementary Video S4). Eight days after this examination, the dog died of old age.

## Discussion

In animals diagnosed with DLSS, the basic treatment consists of abstinence from all physical activities, with the administration of SAID and nonsteroidal anti-inflammatory drugs (NSAID). The application of methylprednisolone epidurally gave good results; it was reported that 53% of animals was totally cured during the follow-up of 1 week to 46 months (9). Also, it was reported that NSAID application for 6 weeks lead to improvement in 50% of treated dogs, which lasted up to 14.5 weeks (10). For dogs that do not respond to this medical treatment, particularly for dogs which are required to work, surgical intervention may be indicated. The surgical management of DLSS includes strategies that are employed with the goal to decompress cauda equina and/or root nerves and/or to stabilize the lumbosacral junction (3).

The most commonly used surgical treatment of decompression is dorsal laminectomy L7 and S1, with dorsal annulectomy, in which the prolonged portion of annulus fibrosus is cut. In studies in which this approach has been applied, different success rate has been reported. In particular, it was shown that the pain relief was detected within 6 weeks in 81% of animals, while positive neurological outcomes were observed in 62.5% of treated dogs within the follow-up of 30 weeks (10); recovery has been reported for 33% of dogs after 2 months following the treatment, with follow-up period of up to 4 years (11); clinical improvement has been reported for 76.8% animals during the follow-up period of 9–41 months (12); and the 53% of dogs were totally cured during the mean follow-up of 30 months (13). This approach, however, often results in a limited decompression effect. Lateral foraminotomy and dorsal decompression technique is also available, although it may increase the instability of L7-S1, as it was shown in 45% of treated patients, during the mean follow-up of 15.2 months (14).

Approaches that aim to stabilize the lumbosacral junction include application of various implants, such as, bone grafting implants of the facets (with or without laminectomy), screw implants (instead of bone grafts), a combination of multiple screw and graft implants that are dorsally connected to each other with bone cement, pedicle screw rod fixation, and transilial bar, for which 53–79% of positive outcomes have been reported during the follow-up period of 6 months to 4 years (3). However, none of these treatments enables optimal stabilization, while, to a greater or lesser extent, all lead to certain complications.

The application of MSCs has emerged as a promising alternative approach to those invasive surgical strategies. Due to the unique properties of MSCs, their application for treating different diseases of the locomotor system, including disc degeneration, has been extensively examined *in vitro* and *in vivo* (6, 15). These cells could be isolated from various tissues, while bone marrow and adipose tissue are most commonly used. The advantage of the latter is easy accessibility of subcutaneous adipose tissue as well as relatively large number of MSCs that it contains (16). It is well known that MSCs can differentiate into various cell types, including chondrocyte lineages, and some studies have shown their capability to differentiate into nucleus pulposus-like cells (17). Other important properties of MSCs include trophic effects that lead to local reduction of inflammation and apoptosis, prevention of fibrosis, stimulation of endogenous regenerative programs, and neovascularization (18). Also, it is important to point out that MSCs can migrate to the point of damage *via* signals sent by tissues affected by ischemia, inflammation, or are otherwise damaged, which is referred to as MSC homing (18–20).

The successful use of AT-MSCs as a regenerative therapy in dogs has mainly been reported for bone and cartilage defects with inflammatory component, such as OA (8, 21–24). Namely, it has been shown that the treatment of canine OA with AT-MSCs leads to a reduction in local inflammation level, and it enables slowdown of degenerative processes and regeneration of damaged articular cartilage (25). Since the similarity between articular cartilage and IVD has been recognized at the morphological, functional, and physiological/pathophysiological levels (26–29), we hypothesized that the positive effects of AT-MSC application, observed in the treatment of canine OA, could also be expected in the treatment of the presented DLSS patient.

Furthermore, we have assumed that autologous AT-MSC transplantation in our DLSS patient near the foramen can lead to a reduction in soft tissue hypertrophy that compresses the root of the nerve. Although soft tissue hypertrophy has been regarded as secondary (30), it is responsible for the appearance of DLSS symptoms and represents a sign of chronic inflammation accompanied with pathological, degenerative processes. Therefore, our assumption was that AT-MSC treatment could facilitate a reduction in the level of local soft tissue chronic inflammation, which would then lead to a decompression of the intervertebral foramen L7-S1.

Positive effects of therapeutic applications of MSCs have been observed in the majority of experimentally induced intervertebral disc degeneration models in small (mouse, rat, rabbit) and large (sheep, dog, minipig) animals (7, 15, 17, 31). In most of these studies, bone marrow MSCs were used, as described for beagle dogs (32). Adipose-MSC transplantation into discs following their nucleotomy has been described for the rat (33) and the dog (34) models.

Even though described *in vivo* studies on induced intervertebral disc degeneration models showed positive effects of MSC application, this therapeutic approach did not lead to improvement when it was used to treat spontaneously occurring IVD degeneration in dogs (4, 5). In these studies, intradiscal injections of 3 × 10^6^ bone marrow-derived MSCs, with or without microcarriers, were used. It was hypothesized that the harsh microenvironment within the IVD, which includes constant loading and low nutrient, oxygen and pH levels, is the reason why MSCs failed to divide and regenerate the damaged structure of IVD (35, 36).

In our case of spontaneous canine IVD degeneration, a different approach of MSC therapy was applied, which proved to be very successful. Namely, we used around 20 times higher number of AT-MSCs (61.2 × 10^6^), which were injected bilaterally in the vicinity of the external aperture of the intervertebral foramen instead of intradiscally. We assumed that this route of application would allow AT-MSCs to exhibit their most important characteristics—trophic and homing effects on all inflamed/edematous/hypertrophied soft intervertebral foramen structures that exert pressure on the nerve root, which would then lead to a decompression of the intervertebral foramen L7-S1 along with the regeneration of the damaged tissues.

It is important to point out that after the application of AT-MSCs, the dog was not subjected to any physiotherapy treatment, as well as that a gradual improvement in clinical outcome measures related to the ability of the dog to walk and trot started to be detected relatively rapidly (30–60 days posttreatment), reaching a marked improvement that was maintained during long-term (4.5 years) follow-up.

## Conclusion

To the best of our knowledge, this is the first case of successful therapy of spontaneously occurring canine DLSS using autologous AT-MSCs that were injected bilaterally in the vicinity of the external aperture of the intervertebral foramen. We believe that the described therapeutic approach represents a contribution to current studies in this field, in both veterinary and human regenerative medicine. Namely, since dogs have been recognized as a superior model compared with other commonly used species for studies of degenerative spinal diseases, DLSS in particular (37, 38), the presented data of safe, minimally invasive AT-MSC treatment with long-lasting positive effects, may shape not only animal but also human DLSS treatment options. The quality of regenerative effect, demonstrated for this DLSS case, should be confirmed in the future by larger, prospective studies that would involve selected canine subjects with spontaneously occurring DLSS.

## Data Availability Statement

The original contributions presented in the study are included in the article/Supplementary Material, further inquiries can be directed to the corresponding author.

## Ethics Statement

The adipose tissue collection and AT-MSC therapy were approved and certified by the dog owner with signed informed consent. The AT collection was performed using anesthetics, and AT-MSC therapy was performed using sedatives, which excluded the pain, suffering, fear, and stress of the animal, following the standards of good veterinary practice, the guidelines of good laboratory practice, the European Parliament Directive, the Council of 22 September 2009 (2010/63/EU), and the European Convention for the protection of vertebrates intended for experimental and other scientific purposes (ETS 170).

## Author Contributions

JM, AF, SP, and GN: conception of the case report. JM: clinical management of the case. VS: AT-MSC isolation and cultivation. JM, SSD, and GN: analysis and interpretation of results. JM, SSD, SP, and GN: writing and editing the manuscript. All authors contributed to the article and approved the submitted version.

## Funding

This work was supported by the Ministry of Education, Science and Technological Development, Republic of Serbia (Grant No. III41004 and 451-03-68/2020-14/ 200042).

## Conflict of Interest

JM was employed by Stem Art Ltd., Belgrade, Serbia. The remaining authors declare that the research was conducted in the absence of any commercial or financial relationships that could be construed as a potential conflict of interest.

## Publisher's Note

All claims expressed in this article are solely those of the authors and do not necessarily represent those of their affiliated organizations, or those of the publisher, the editors and the reviewers. Any product that may be evaluated in this article, or claim that may be made by its manufacturer, is not guaranteed or endorsed by the publisher.

## Supplementary Material

Supplementary Video S1 A short video from the period just before the initial veterinary evaluation (baseline)—provided by the owner of the dog.

Supplementary Video S2 Video from the period 3 years post-AT-MSC treatment—provided by the veterinarian.

Supplementary Video S3 Video from the period 3 years post-AT-MSC treatmentprovided by the veterinarian.

Supplementary Video S4 Video from the period 4.5 years post-AT-MSC treatment—provided by the veterinarian.

## Abbreviations

AT: adipose tissue
DLSS: degenerative lumbosacral stenosis
IVD: intervertebral disc
MRI: magnetic resonance imaging
MSCs: mesenchymal stem cells
OA: osteoarthritis
PE: physical examination.
